# Influence of Radio-Activity on Plant Growth

**Published:** 1914-02

**Authors:** 


INFLUENCE OF RADIO-ACTIVITY ON PLANT GROWTH. The November, 1913 issue of *La Radiumculture* contains several illustrated articles, showing the increased growth of beets, potatoes, Brussels sprouts etc., under the influence of radio-active powders, as compared with controls.

